# TIM-1 As a Signal Receptor Triggers Dengue Virus-Induced Autophagy

**DOI:** 10.3390/ijms20194893

**Published:** 2019-10-02

**Authors:** Li-Wei Chu, Chia-Jui Yang, Kuan-Jen Peng, Pei-Ling Chen, Shuu-Jiun Wang, Yueh-Hsin Ping

**Affiliations:** 1 Institute of Biophotonics, National Yang-Ming University, Taipei 11221, Taiwan; nealchu125@gmail.com; 2 Brain Research Center, National Yang-Ming University, Taipei 11221, Taiwan; sjwang@vghtpe.gov.tw; 3 Faculty of Medicine, National Yang-Ming University, Taipei 11221, Taiwan; yangcj1206@gmail.com; 4 Department of Internal Medicine, Far Eastern Memorial Hospital, New Taipei City 22060, Taiwan; 5 Department and Institute of Pharmacology, National Yang-Ming University, Taipei 11221, Taiwan; kjpeng8@gmail.com (K.-J.P.); s904158@gmail.com (P.-L.C.); 6 Department of Neurology, Neurological Institute, Taipei Veterans General Hospital, Taipei 11217, Taiwan

**Keywords:** dengue virus, autophagy, TIM-1, p85, rab5, early endosome

## Abstract

Dengue virus (DENV) infection triggers the activation of autophagy to facilitate the viral replication cycle from various aspects. Although a number of stimulators are proposed to activate autophagy, none of them appears prior to the uncoating process. Given that T-cell immunoglobulin and mucin domain 1 (TIM-1) receptor is a putative DENV receptor and promotes apoptotic body clearance by autophagy induction, it raises the possibility that TIM-1 may participate in the activation of DENV-induced autophagy. In this study, confocal images first revealed the co-localization of TIM-1 with autophagosomes in DENV-induced autophagy rather than rapamycin-induced autophagy, suggesting the co-transportation of TIM-1 with DENV during infection. The treatment of siRNA to knockdown TIM-1 expression in DENV-infected GFP-microtubule-associated protein light chain 3 (LC3)-Huh7.5 cells revealed that TIM-1 is required not only for DENV cellular internalization but also for autophagy activation. Furthermore, knockdown p85, a subunit of phosphoinositide 3-kinases (PI3Ks), which is co-localized with TIM-1 at rab5-positive endosomes caused the reduction of autophagy, indicating that TIM-1-mediated DENV-induced autophagy requires p85. Taken together, the current study uncovered TIM-1 as a novel factor for triggering autophagy in DENV infection through TIM-1-p85 axis, in addition to serving as a DENV receptor.

## 1. Introduction

Dengue virus (DENV), an RNA virus belonging to the *flaviviridae* family, which includes emerging and reemerging pathogens such as Zika virus (ZIKV), Japanese encephalitis virus (JEV), and West Nile virus (WNV), causes the most prevalent arthropod-born viral disease, with an estimated one hundred million symptomatic cases every year around the world [1]. DENV infection causes human diseases with a wide spectrum of clinical symptoms, ranging from asymptomatic infection or self-limited febrile illness named Dengue fever (DF) to life-threatening diseases including Dengue hemorrhagic fever (DHF) and Dengue shock syndrome (DSS) [2,3,4]. Currently, specific treatments for DENV are lacking. There is still an urgent need for anti-Dengue agents to prevent or treat DENV infections. As a result, more detail insights into DENV biology and Dengue-host interactions are necessary. DENV infection is a complicated and multifaceted process. DENV initiates infection of a permissive cell through binding of viral E protein with cellular receptors [5,6]. After the interaction of receptors, DENV virus particles are internalized into cells through the clathrin-mediated endocytosis pathway [7,8,9]. To release the viral RNA genome, DENV virions undergo an acid-induced conformational change and membrane fusion. Newly synthesized viral proteins generated near the endoplasmic reticulum (ER) promote replication of the viral RNA genome, induction of membrane rearrangement, and assembly of new viral particles [10,11]. To facilitate the process of DENV replication, DENV not only interacts with various cellular components, but also triggers various host responses, such as autophagy.

Autophagy is a catabolic process that degrades damaged or excess intracellular components to recycle nutrients for regeneration of energy and cellular organelles and is essential to maintain cellular as well as organismal homeostasis [12,13]. Autophagy also play a critical role in the cellular defense mechanism against viral infection by either directly eliminating the pathogens or indirectly facilitating host immune responses [14,15,16]. Some viruses, such as sindbis virus, herpes simplex virus-1 (HSV-1), murine gamma-herpesvirus 68 (MHV-68), and vesicular stomatitis virus, have successfully evolved strategies to block autophagy activation for survival [17,18,19,20]. Others have developed different strategies to utilize autophagy for promoting the viral replication process [14,16,21,22,23]. DENV infection activates autophagy and subverts the autophagic machinery to promote robust viral replication and intracellular spreading in different ways [24,25,26,27,28,29]. Although the activation of autophagy by DENV infection has been clearly demonstrated, little is known about how DENV initiates this process. From what little we know, DENV-induced autophagy can be triggered by various signals, such as viral non-structure protein 4A (NS4A), non-structure protein 4B (NS4B) protein, AMP-activated protein kinase (AMPK), and ER stress, which apparently appear in the later phase of infection [16,25,30,31,32]. Our recent study showed that autophagy is activated at 15 min post-infection [9], suggesting an early triggering signal pathway of autophagy prior to the viral uncoating process.

T-cell/transmembrane immunoglobulin and mucin domain protein-1 (TIM-1), a type I transmembrane glycoprotein, contains an extracellular domain composed of an N-terminal immunoglobulin variable (IgV)-like domain followed by a glycosylated mucin domain, a single transmembrane domain, and a short cytoplasmic tail with tyrosine phosphorylation motifs [33]. TIM-1 is a receptor of phosphatidylserine (PtdSer), a signal of cell death exposed on the outer leaflet of the apoptotic cell membrane [34,35]. The binding of TIM-1 with PtdSer on apoptotic cells through its metal ion-dependent ligand binding site (MILIBS) within IgV domain promotes apoptotic clearance [36,37]. TIM-1 is also known as Hepatitis A virus (HAV) cellular receptor 1 (HAVCR1), which was first identified as an HAV cellular receptor [38]. Moreover, growing evidence has proven TIM-1 to be a cellular receptor which facilitates viral infection, and existent in a number of various viruses, including Ebola virus (EBOV), Marburg virus (MARV), Lassa virus, HAV, Hepatitis C virus (HCV), JEV, and DENV [39,40,41,42,43,44,45,46]. Several findings have elucidated that TIM-1-mediated enhancement of infection mainly depends on the association of PtdSer exposed on the viral envelop [42,47]. Amara and colleagues recent revealed that DENV infection is mediated by TIM-1 in a PtdSer-dependent manner and ubiquitination of TIM-1 is required for DENV cellular entry [40,45].

Given that the activation of autophagy by DENV is prior to the uncoating process, TIM-1 is recognized as a DENV entry receptor. Since TIM-1-mediated phagocytosis of apoptotic bodies induces autophagy signaling for apoptotic clearance [9,40,48], we hypothesized that TIM-1 mediates DENV-induced autophagy to facilitate DENV production. In this study, we first demonstrated that TIM-1 is co-localized with the GFP-microtubule-associated protein light chain 3 (LC3)-positive autophagic vesicle in DENV-infected cells, but not in cells treated with rapamycin, a stimulator of autophagy. The treatment of siRNA against TIM-1 and p85, a subunit of phosphoinositide 3-kinases (PI3Ks), reduced the formation of GFP-LC3 punta as well as the production of DENV particles. Confocal images revealed co-localization of TIM-1 with p85 at rab5-positive endosomes in DENV-infected cells, indicating the requirement of PI3Ks for TIM-1-mediated DENV-induced autophagy. Our results elucidated dual roles of TIM-1 of facilitating viral entry and activating autophagy during DENV infection.

## 2. Results

### 2.1. TIM-1 Required for the Activation of DENV-Induced Autophagy

**Figure 1:**
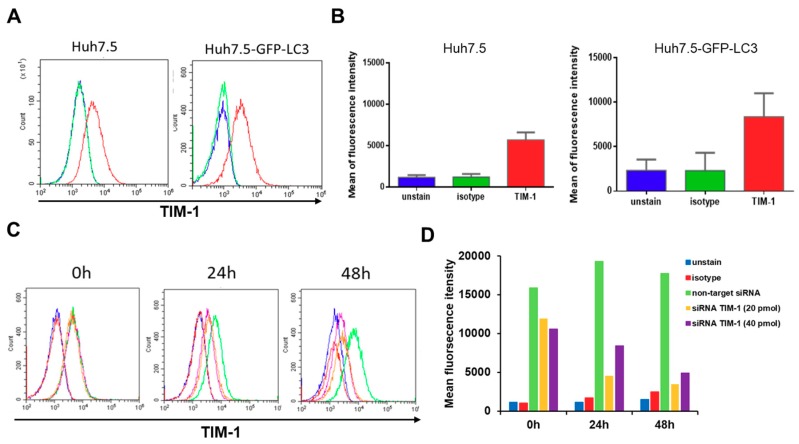
The expression of T-cell/transmembrane immunoglobulin and mucin domain protein-1 (TIM-1) and the treatment of siRNA against TIM-1 in Huh7.5 and GFP-microtubule-associated protein light chain 3 (LC3)-Huh7.5 cells. (**A**) TIM-1 expressed on the surface of Huh7.5 and GFP-LC3-Huh7.5 cells (red line). The green line represents cell staining with a control antibody and the blue line represents unstained cells. (**B**) The quantification of the histogram depicted the mean fluorescence intensity of each group from (**A**). (**C**) We confirmed by flow cytometry the reduced expression of TIM-1 after using specific siRNA (si-TIM-1). Representative experiments of the transfection of GFP-LC3-Huh7.5 with non-targeting siRNA (blue) and si-TIM1 with 20 pmol (orange) and 40 pmol (purple) were displayed, respectively. Two additional control groups, including unstained and isotype, were marked in blue and red. (**D**) The quantification of the histogram presented the mean fluorescence intensity of each group from (**C**).

To reveal the roles of TIM-1 on DENV-induced autophagy, immunofluorescence staining first confirmed the expression of TIM-1 in Huh7.5 cells, which is a DENV highly permissive cell lines derived from cellular hepatocellular carcinoma [10,49], and GFP-LC3 stably expressing Huh7.5 cells (GFP-LC3-Huh7.5). Both cell lines were stained with phycoerythrin (PE)-conjugated anti-TIM-1 antibodies and fluorescence signals were detected and analyzed by flow cytometry. The histograms of flow cytometry depicted a right-shift peak of PE signals in the anti-TIM-1 antibody staining group, compared to that in the unstaining and isotype-staining groups served as negative controls (Figure 1A). The quantitative results of the mean fluorescence intensity (MFI) revealed at least five-fold stronger signals in the anti-TIM-1 antibody staining group than the control groups (Figure 1B).

These results suggested the expression of TIM-1 in both Huh7.5 and GFP-LC3-Huh7.5 cell lines. Moreover, to investigate the roles of TIM-1 in the activation of DENV-induced autophagy, we also generated TIM-1-knockdowned GFP-LC3-Huh7.5 cells by a siRNA (si-TIM-1) treatment. Dynamic expression of TIM-1 of siRNA-treated GFP-LC3-Huh7.5 cells was examined and quantified by flow cytometry. In comparison with antibody-free, isotype control, and non-target siRNA control (si-Control) groups, the expression of TIM-1 declined as time of treatment in si-TIM-1 groups increased (Figure 1C,D), indicating the treatment of si-TIM-1 in GFP-LC3-Huh7.5 cells can specifically repress the expression of TIM-1.

**Figure 2:**
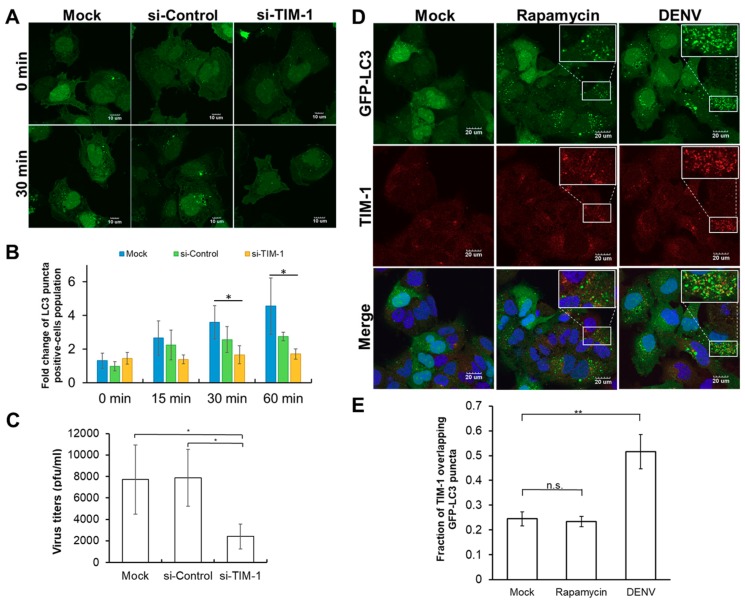
The requirement of TIM-1 for Dengue virus (DENV)-induced autophagy and virus production. (**A**) The formation of autophagosomes (green) in GFP-LC3-Huh7.5 cells treated either with si-control or si-TIM-1 prior to DENV infection at a multiplicity of infection (MOI) of five were visualized by Olympus FluoView 1000 confocal microscopy at 0 and 30 min post-infection, respectively. The scale bar represents 10 μm. (**B**) The effects of either si-control (red) or si-TIM-1 (yellow) treatment on the formation of autophagy in GFP-LC3-Huh7.5 cells were determined by the percentage of autophagy-positive cells. siRNA-free group indicates as mock (blue). The error bars represent standard deviations from three independent experiments (>200 cells). * *p* < 0.05. (**C**) The effects of TIM-1 on DENV production were examined. GFP-LC3-Huh7.5 cells were treated with siRNA prior to DENV infection. The titers of the extracellular virus were measured by a plaque assay 24 h post-infection. The means of three independent experiments performed in triplicate are shown. * *p* < 0.05. (**D**) Distribution of TIM-1 (red) and GFP-LC3 punta (green) in GFP-LC3-Huh7.5 cells treated either with rapamycin or DENV infection for an hour were determined by confocal microscopy (Olympus FV-10i). The insets showed more colocalization of TIM-1 with LC3 puncta in DENV-infected cells rather than in rapamycin-treated cells. Scale bar, 20 μm. (**E**) The colocalization of TIM-1 with GFP-LC3 punta was quantitatively analyzed by Mander’s coefficient from three independent experiments (>25 cells). n.s. means non-significant; ** *p* < 0.01.

To confirm TIM-1 is indeed required for DENV-induced autophagy, the numbers of GFP-LC3 punta that represents the formation of autophagosomes in TIM-1-knockdowned cells was examined. GFP-LC3-Huh7.5 cells were infected by DENV at a multiplicity of infection (MOI) of five in the absence or presence of siTIM-1. Apparently, compared to siRNA-free (mock) and si-Control groups, the treatment of si-TIM-1 inhibited the formation of GFP-LC3-positive autophagosomes in GFP-LC3-Huh7.5 cells at 30 min post-infection (Figure 2A). The quantification results of time course experiments revealed that the numbers of GFP-LC3-positive punta cells were elevated in either mock or si-Control-treated DENV-infected cells at 15, 30 and 60 min post-infection (Figure 2B). In contrast, the siTIM1 treatment effectively suppressed the formation of GFP-LC3-positive autophagosomes in DENV-infected cells (Figure 2B). Furthermore, given that autophagy promotes DENV production [9,29,50], the effect of the siTIM1 treatment on viral production was examined by the plaque assay. Significantly, the titer of newly synthesized DENV particles decreased in the presence of si-TIM-1 (Figure 2C). This shows that TIM-1 triggers autophagy in DENV infection, which would in turn affect viral production facilitation. As previous studies have shown that TIM-1 also serves as an entry receptor of DENV [40], this implies that it might in fact have a dual role in DENV infection. Accordingly, TIM-1 has been demonstrated to be an entry receptor of DENV because genetic ablation of TIM-1 inhibits DENV infection [40]. Taken together, these results indicated that TIM-1 might exert dual roles, facilitating DENV entry and triggering autophagy, in DENV infection.

Because DENV binds with TIM-1 to initiate infection and DENV particles are translocated into autophagosomes after internalization [9,40], we speculated that TIM-1 is located in autophagosomes after DENV infection. To investigate this possibility, the images of GFP-LC3 punta, which represent the formation of autophagosomes, and TIM-1 in GFP-LC3-Huh7.5 cells that were exposed to DENV at a MOI of 10 for one hour were visualized by fluorescence microscopy. The numbers of GFP-LC3 punta increased significantly by either the treatment of rapamycin or DENV in GFP-LC3-Huh7.5 cells, suggesting the activation of autophagy (Figure 2D, Top panel). By merging the signals of GFP-LC3 and immunofluorescence images of TIM-1 (Figure 2D, middle panel), we elucidated the colocalization of TIM-1 with autophagosomes. Apparently, TIM-1 signals overlapped with GFP-LC3 punta when cells were infected with DENV instead of treated with rapamycin (Figure 2D, bottom panel). The quantification of TIM-1 colocalized with autophagosomes was determined by Mander’s coefficient of TIM-1 signals overlapping GFP-LC3 punta. As shown in Figure 2E, the value of Mander’s coefficient in the DENV group is 0.51, which is significantly higher than that in either mock or rapamycin-treated group. In summary, these results indicated that TIM-1 is specifically located within autophagosomes in DENV-induced autophagy rather than in rapamycin-induced autophagy.

### 2.2. The Colocalization of TIM-1 with DENV Particles in Autophagosomes

**Figure 3:**
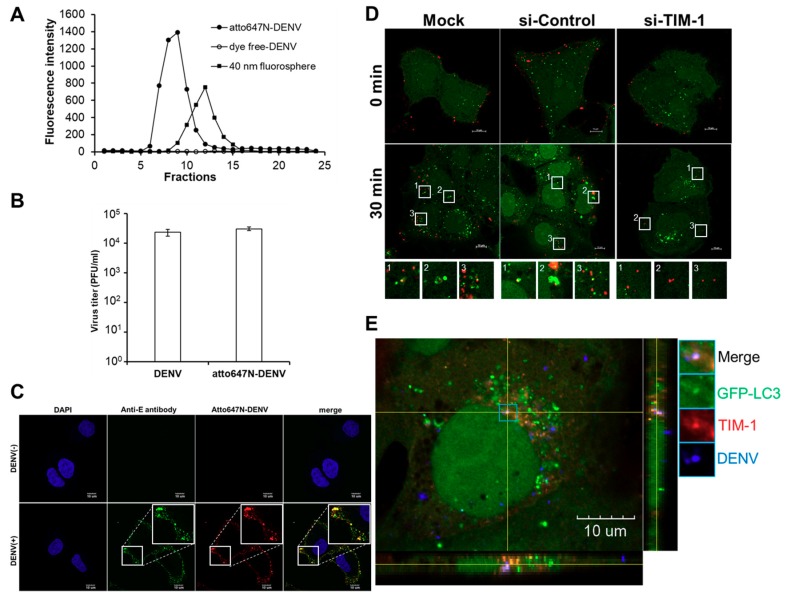
Colocalization of TIM-1 with DENV particles and autophagosomes. (**A**) The fluorescence intensity profiles of elution from a Sephadex G-25 size-exclusion column was displayed. Atto647N-labeled DENV particle was purified within the fraction layer 7 to 11 from gel filtration (solid circle). No fluorescence signal was detected in Atto647N-free DENV (open circles) within the same fraction layers. The 40 nm fluorescence microspheres were used as a size marker (solid square). (**B**) Plaque assays were performed to determine the infectivity of DENV particles incubated with Atto647N. Atto647N-free DENV (DENV) was used as a control. Virus particles were purified from the same fractions of the Sephadex G-50 column. The results were determined by at least three independent experiments. (**C**) Atto647N-DENV particles were visualized by an anti-E antibody. Atto647N-DENV (MOI of 20) attached to the surface of Huh7.5 cells at 4 °C for 30 min were recognized by an anti-E antibody. The insets showed the localization of Atto647N-DENV signals coincides with DENV E protein. The scale bar represents 10 μm. (**D**) The formation of autophagosomes (green) and the distribution of Atto647N-DENV particles (red) were displayed by confocal imaging (Olympus FluoView 1000). GFP-LC3-Huh7.5 cells were treated either with si-control or si-TIM-1 prior to DENV infection at a MOI of 10. The confocal images were acquired by the Olympus FluoView 1000 confocal microscopy at 0 and 30 min post-infection, respectively. The insets showed no colocalization of Atto647N-DENV signals with LC3 puncta in si-TIM-1-treated cells. The scale bar represents 10 μm. (**E**) The three-dimensional reconstruction of the Z-stacked deconvolved image showed the co-localization of TIM-1 (red) with DENV particles (blue) on an autophagosome (green) in a GFP-LC3-Huh7.5 cell one hour post-infection. The scale bar represents 10 μm.

Since DENV infection specifically enhanced co-localization of TIM-1 and autophagosomes (Figure 2D,E), we would like to investigate the possibility that DENV particles and TIM-1 are co-localized in autophagosomes by using the single-virus imaging analysis. To visualize single DENV particles directly, DENV particles were first labeled with a fluorescence dye, atto647N, by conjugating atto647N-NHS ester with the amino group of the viral envelope protein and purified through a Sephadex G-25 size-exclusion column. As the diameter of a DENV particle is 50 nm, the fluorescent signals of atto647N existent from fraction 7 to fraction 11 (Figure 3A, solid circles) indicated atto647N-labeled DENV particles (atto647N-DENV) when compared to the fractions containing the 40 nm fluorosphere (Figure 3A, solid squares). No fluorescence signal was detected within the same range of fractions from the dye-free DENV group (Figure 3A, open circles). These results elucidated that DENV particles were successfully conjugated with atto647N. The infectivity of atto647N-DENV particles was then determined by the plaque assay. Both dye-free DENV and atto647N-DENV presented similar virus titers, suggesting that the atto647N labeling procedure showed no significant influence on DENV infectivity (Figure 3B). To visualize atto647N-DENV particles, Huh7.5 cells were incubated with atto647N-DENV cells at 4 °C for 30 min. The surrounding distribution pattern of atto647N signals indicated the binding of DENV on the surface of Huh7.5 cells (Figure 3C). We further detected DENV virion by using an anti-DENV E protein antibody. Immunofluorescent staining revealed that the atto647N signals and the anti-DENV E protein antibody overlapped, suggesting atto647N spots can represent DENV virion images (Figure 3C). These results confirmed the generation of infectious atto647N-DENV particles.

To confirm whether atto647N-DENV particles induce autophagy and colocalize with autophagosomes in the absence or presence of si-TIM-1, GFP-LC3-Huh7.5 cells, pre-treated with either si-control or si-TIM-1 for 48 h were incubated with atto647N-DENV cells at an MOI of 10 at 37 °C for 30 min. The confocal microscopy images showed that not only an increase in the number of GFP-LC3-positive vesicles was detected, which represents the formation of autophagosomes, but also the events of atto647N-DENV co-localization with autophagosomes elevated in both mock (siRNA-free) and si-control groups, as compared to those at the initial time point of infection (Figure 3D). This was in line with our previous study [9]. In contrast, the number of internalized DENV particles (red) and the formation of GFP-LC3-positive autophagosomes (green) in si-TIM-1-treated Huh7.5 cells apparently decreased (Figure 3D). In addition, triple-fluorescence imaging from confocal Z-stack images and cross-sectional analyses using fluorescence intensity profiling displayed spatial coincidence of GFP-LC3 punta (green), TIM-1 (red), and atto647N-DENV (blue) in GFP-LC3-Huh7.5 cells (Figure 3E), suggesting TIM-1 is co-localized with the DENV particle in an autophagosome. To sum up, these results elucidated that TIM-1 plays dual roles of promoting viral entry and triggering autophagy during the early phase of DENV infection.

### 2.3. The Colocalization of p85 with TIM-1 in rab5-Positive Endosomes

**Figure 4:**
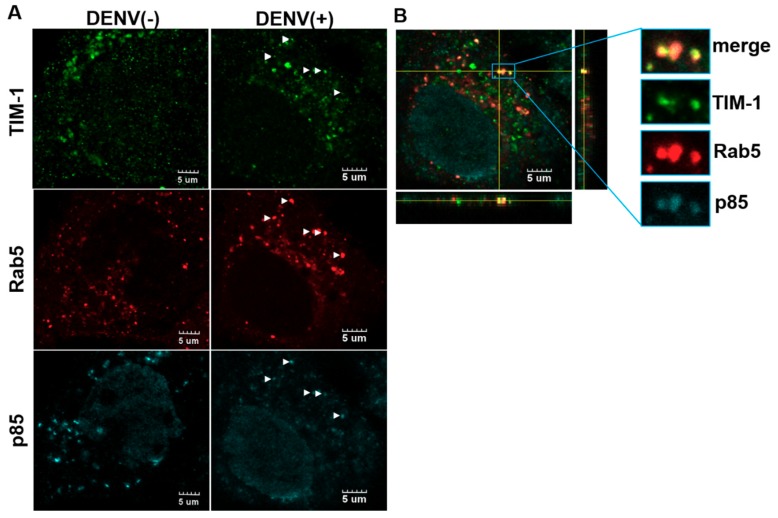
The colocalization of TIM-1 with p85 at Rab5-positive endosomes by DENV infection. (**A**) The cellular location of TIM-1 (green), p85 (cyan), and Rab5-positive endosome (red) were visualized in Huh7.5 cells transfected with a Rab5-DsRed plasmid in the absence or presence of DENV infection at a MOI of 10 for 30 min post-infection. Fluorescence images were acquired using an Olympus FluoView 1000 confocal microscopy. White arrowhead indicated triple-colocalization. The scale bar represents 5 μm. (**B**) The three-dimensional reconstruction of the Z-stacked deconvolved image showed the colocalization of TIM-1 with p85 at Rab5-positive endosomes. In fluorescence intensity profiling, the signal distribution of TIM-1, p85 and Rab5-positive endosomes were indicated in green, cyan, and red, respectively.

Given that TIM-1 interacts with p85, the PI3K adaptor, resulting in autophagy induction [48], we proposed that p85 may be a downstream signal molecule for TIM-1-mediated autophagy activation induced by DENV. In addition, as DENV particles are internalized with TIM-1 and located in Rab5-positive endosomes after clathrin-mediated endocytosis [7,40], it is highly possible that TIM-1 is co-localized with p85 in endosomes. To validate this possibility, Huh7.5 cells were transfected with Rab5-DsRed plasmids prior to DENV infection at a MOI of 10. The location of TIM-1, p85 and Rab5-positive endosomes were detected at 30 min post-infection. Compared to the virus-free control group (mock), DENV infection enhanced the co-localization of TIM-1, p85 in Rab5-positive endosomes (Figure 4A). Fluorescence intensity profiling from cross sectional analyses of confocal Z-stack images depicted spatial coincidence of GFP-TIM-1 (green), Rab5-positive endosomes (red), and p85 (cyan) in Huh7.5 cells (Figure 4B). These results supported that DENV infection enhances the association of TIM-1 with p85 at endosomes at the early phase of DENV-infection.

### 2.4. p85 Knockdown Attenuates DENV-Induced Autophagy and Virus Production

**Figure 5:**
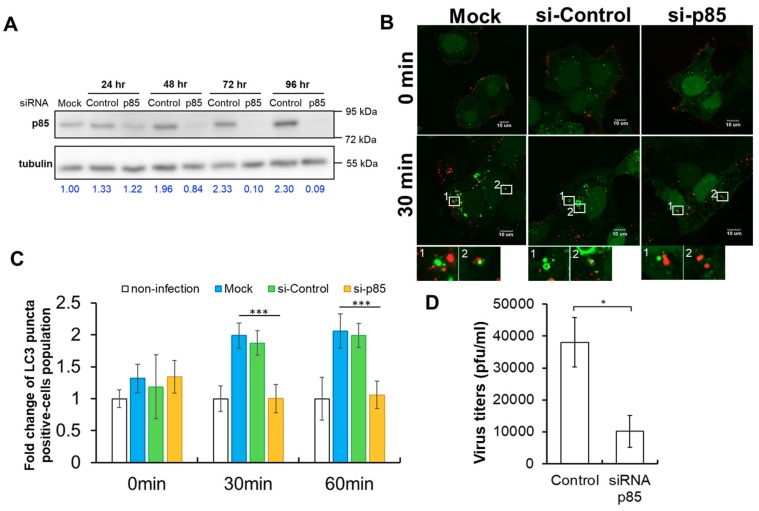
The involvement of p85 in TIM-1-mediated DENV-induced autophagy and virus production. (**A**) The expression of p85 in GFP-LC3-Huh7.5 cells transfected with either non-targeting siRNA or siRNA against p85 was analyzed by a Western blot assay at 24, 48, and 72 h post-transfection. Mock indicated the parental GFP-LC3-Huh7.5 cells without siRNA transfection. Numbers at the bottom represent the fold changes of the expression of p85 normalized with one in the mock group. (**B**) The formation of autophagosomes (green) and the distribution of Atto647N-DENV particles (red) in GFP-LC3-Huh7.5 cells treated either with si-control or si-p85 prior to DENV infection at a MOI of 10 were visualized by Olympus FluoView 1000 confocal microscopy at 0 and 30 min post-infection, respectively. The insets showed no colocalization of Atto647N-DENV signals with LC3 puncta in si-p85-treated cells. The scale bar represents 10 μm. (**C**) The effects of either si-control (red) or si-p85 (yellow) treatment on the formation of autophagy in GFP-LC3-Huh7.5 cells was determined by the percentage of autophagy-positive cells. The siRNA-free group is indicated as mock (blue). GFP-LC3-Huh7.5 cells without both DENV infection and siRNA transfection were indicated as non-infection (white). The error bars represent standard deviations from three independent experiments (>200 cells). *** *p* < 0.001. (**D**) The effects of p85 on DENV production were examined. GFP-LC3-Huh7.5 cells were treated with si-p85 prior to DENV infection. The titers of the extracellular virus were measured by a plaque assay 48 h post-infection. The means of three independent experiments performed in triplicate are shown. * *p* < 0.05.

To further elucidate the function of p85 in DENV-induced autophagy, we examined the formation of GFP-LC3-positive punta in GFP-LC3-Huh7.5 cells treated with siRNA against p85 (si-p85). The expression of p85 of siRNA-treated GFP-LC3-Huh7.5 cells was examined and quantified. Western blotting assays depicted clearly that the expression of p85 was reduced by si-p85 (Figure 5A). Compared to those in the non-target siRNA control group (si-Control), quantification data showed more than 50% knockdown efficiency of p85 expression after 48 h post-transfection. To validate the requirement of p85 in the activation of DENV-induced autophagy, GFP-LC3-Huh7.5 cells were infected by atto647N-DENV at a MOI of 10 in the absence or presence of si-p85 and the formation of GFP-LC3-positive autophagosomes was examined. Apparently, compared to siRNA-free (mock) and si-Control groups, the treatment of si-p85 inhibited the formation of GFP-LC3-positive autophagosomes but not the internalization of DENV particles in GFP-LC3-Huh7.5 cells (Figure 5B). Quantification of the time course results revealed that the numbers of GFP-LC3-positive autophagosomes were elevated in either siRNA-free or si-Control-treated DENV-infected cells at 30 and 60 min post-infection (Figure 5C). In contrast, si-p85 treatment effectively suppressed the formation of GFP-LC3-positive autophagosomes in DENV-infected cells (Figure 5C). Furthermore, the effect of the si-p85 treatment on the new synthesis of DENV particles was examined by the plaque assay. The production of DENV particles was significantly repressed in the presence of si-p85 (Figure 5D). Taken together, these results confirmed that p85 is required for DENV-induced autophagy and contributes to DENV production.

## 3. Discussion

**Figure 6:**
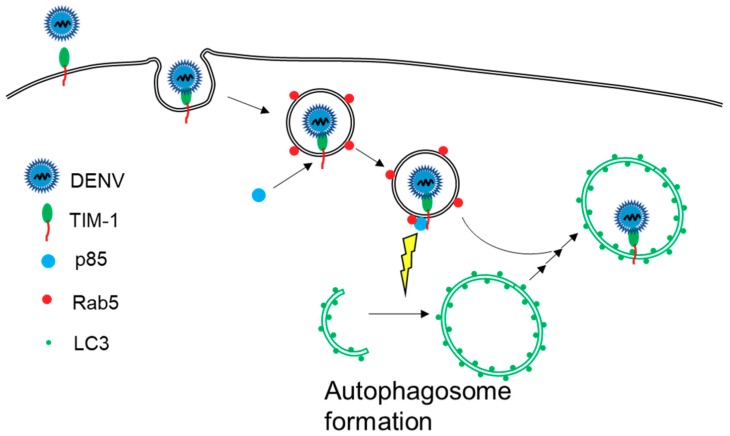
The scheme of TIM-1-mediated DENV-induced autophagy activation. DENV binds with TIM-1 to internalize into cells. Following receptor-mediated endocytosis, DENV delivers with TIM-1 in the Rab5-endosome and recruits p85 association. TIM-1 interacts with p85 and Rab5 to enhance autophagosome formation. Furthermore, DENV-contained endosomes deliver DENV to autophagosomes.

The present study uncovers a novel role of TIM-1 during DENV infection in which TIM-1 specifically triggers DENV-induced autophagy through the PI3K pathway at early phase of infection. We, thus, would like to define an unprecedented triggering mechanism of autophagy by DENV infection as illustrated in Figure 6. To initiate the infection process, a DENV particle is first recognized by TIM-1 and internalized into a Rab5-positive endosome through clathrin-mediated endocytosis [7,40]. Next, p85 is recruited to TIM-1-positive endosomes, which activates autophagy to form autophagosomes through the PI3K pathway. Finally, the DENV particle is transported into an autophagic vesicle with TIM-1 for further processing in the viral life cycle to produce new viral particles. Recognizing that TIM-1 is not only a DENV receptor, but also a key cellular factor triggering the activation of DENV-induced autophagy through the PI3K pathway provides a mechanistic understanding of the dual roles of TIM-1 in facilitating DENV production.

Autophagy is initially activated by DENV in the early phase of infection before the viral uncoating process [9]. Although several factors including DENV NS4A/NS4B, virus-induced ER stress, and AMPK have been proposed to be required for autophagy [16,25,30,31,32], none of them appears prior to the viral uncoating process, suggesting an unidentified mechanism of activated autophagy during the early phase of DENV infection. DENV has evolved an elegant strategy, named viral apoptotic mimicry, in which it manipulates host cells for cellular entrance by the exposure of PtdSer on the surface [49]. DENV mimics apoptotic cells by exposing PtdSer at the viral membrane, to facilitate the binding of TIM-1 [45], which functions as an authentic DENV cellular receptor to promote DENV entrance and plays an active role in virus endocytosis [40]. As TIM-1 binds with PtdSer on apoptotic cells to promote apoptotic clearance by activating autophagy [48], we speculated that TIM-1 might mediate DENV-induced autophagy. Using siRNA to knockdown TIM-1 not only reduced DENV internalization (Figure 3D), which is consistent with previous studies [45], but also significantly suppressed DENV-induced activation of autophagy (Figure 2A,B). Both of the effects may contribute to the reduction of DENV virions production (Figure 2C) since DENV infection triggers autophagy to facilitate viral production [9,29,50]. This study thus not only corroborated with previous findings with regards to TIM-1 acting as an DENV entry receptor [40], but also elucidated a novel signaling role of TIM-1 as a trigger to activate autophagy in DENV infection.

In the current study, we revealed that treatments of both DENV and rapamycin can trigger autophagy, but the colocalization of TIM-1 with autophagosomes is only detected in DENV-induced autophagy (Figure 1C), suggesting that the activation of DENV-induced autophagy may be independent of the conventional pathways in which DENV production can be enhanced by rapamycin treatments [9,29,51,52]. In contrast to TIM-1 that activates autophagy right after cellular internalization of DENV, other autophagy activators, such as DENV NS4A/B proteins, ER stress, and AMPK appear after viral translation [16,25,30,31,32], suggesting the existence of multiple activators of autophagy during DENV infection. This implies that autophagy triggered by various activators plays different roles to facilitate DENV production. For instance, DENV infection activates AMPK for viral replication as well as lipophagy induction but not basal autophagy [32]. Moreover, one recent study reported that autophagy is required for the post-RNA replication process during DENV infection in HeLa cells [53]. As HeLa cells are TIM-1-negative cells [45], we speculated that autophagy cannot benefit from DENV replication in the early phase of infection in a TIM-1-absence cell line. In contrast, DENV presents a higher infectious ability in both Huh7.5 and A549 cells of TIM-1 positive cell lines [45]. Knockdown TIM-1 expression in Huh7.5 cells suppressed autophagy and reduced DENV production (Figure 2). The results indicated that TIM-1 promotes DENV replication by activating autophagy in the early phase of infection. Moreover, there are non-autophagic functions of autophagy-relevant proteins, such as endocytosis, LC3-associated phagocytosis, and cellular transportation [54]. It would be worthy to investigate how DENV selects and controls these different pathways for viral production and whether there are additional host factors, such as autophagy-relevant proteins, involved in DENV replication.

In addition to TIM-1, the current study also revealed the requirement of p85, a regulatory subunit of PI3Ks, for DENV-induced autophagy (Figure 5C). The initiation of autophagy is highly regulated by a group of phosphoinositides generated by PI3Ks [55]. p85 participated in KIM-1/TIM-1 phosphorylation-mediated signaling [56] and regulates starvation-induced autophagy through IKK [57]. Our results also depicted the co-localization of p85 with TIM-1 in DENV-infected cells (Figure 4A). These results are consistent with the previous finding that KIM-1/TIM-1 interacts with p85 to induce autophagy for the clearance of apoptotic cells in the KIM-1/TIM-1-mediated phagocytosis [48]. Moreover, p110β, a catalytic subunit of PI3Ks, associates with Rab5 and enhances the rab5-Vps34 interaction to promote autophagy [55]. Given that DENV particles are internalized with TIM-1 and located in the rab5-positive endosome after clathrin-mediated endocytosis and TIM-1 is co-localized with p85 at endosomes (Figure 4) [7,40], we would like to propose a novel TIM-1-p85 signaling axis model for activating DENV-induced autophagy. After cellular internalization with DENV, TIM-1 interacts with p85 in early endosomes and recruits PI3Ks to enhance the rab5-Vps34 interaction, promoting activation of autophagy. Further studies are thus needed in order to address the following issues: Whether p85 dynamically associates with DENV-containing vesicles, whether interaction of rab5 with Vps34 is required for DENV-induced autophagy, and whether there are additional host factors involved in DENV-induced autophagy.

Given that an increase in cellular autophagy facilitates the production of many viruses, a common evolutionary selection exists among various viruses to trigger or activate autophagy [16,22]. Additionally, TIM-1 is recognized as a cellular receptor of various viruses to facilitate viral infection [39,40,41,42,43,44,45,46]. In this study, we showed a novel role of TIM-1 to activate autophagy through the TIM-1-p85 signaling axis in the early phase of DENV infection, suggesting the dependence of TIM-1 on the activation of autophagy of DENV infection. Because TIM-1 is recognized as a therapeutic target in cancers, kidney injury, and immune diseases [58,59,60], we believe that developing potent TIM-1 inhibitors might be a promising possibility for successful anti-DENV and anti-viral drugs in the future.

## 4. Materials and Methods

### 4.1. Cell Culture

Huh7.5 cells were maintained in Dulbecco’s modified Eagle’s medium (DMEM, Gibco, 11995, Grand Island, NY, USA) supplemented with 10% fetal bovine serum (FBS, HyClone, SH3007, South Logan, UT, USA), 1× nonessential amino acids (NEAA, Gibco, 11140, Grand Island, NY, USA), and 1% penicillin / streptomycin (P/S, Gibco, 15070, Grand Island, NY, USA). The stable clone GFP-LC3-Huh7.5 cells which stably expressed GFP-LC3 were generously provided by Michael M.C. Lai (Institute of Molecular Biology, Academia Sinica, Taipei, Taiwan) [61], and were maintained in DMEM (Gibco, 11995, Grand Island, NY, USA) supplemented with 10% FBS, 1× NEAA, 1% P/S, and 4 μg/mL blasticidin S HCl (BSD, Invitrogen, A1113903, Carlsbad, CA, USA). The baby hamster kidney-21 (BHK-21) cells used to quantify virus titer were maintained in DMEM (Gibco, 12100, Grand Island, NY, USA) supplemented with 10% FBS and 1% P/S. C6/36 cells derived from Aedes albopictus larva body for DENV propagation, which were maintained in the Roswell Park Memorial Institute 1640 (RPMI 1640, Gibco, 31800, Grand Island, NY, USA), supplemented with 10% FBS, 1× NEAA, 1% P/S, and 1mM sodium pyruvate (Gibco, 11360-070, Grand Island, NY, USA). Only C6/36 cells were incubated at 28 °C with 5% CO_2_. The other cell lines were incubated at 37 °C with 5% CO_2_.

### 4.2. DENV Amplification

DENV strain 16681 serotype 2 was propagated in C6/36 cells. C6/36 cells were grown until the confluent monolayer is formed and infected with DENV at MOI 0.02 in 2 mL serum free RPMI 1640 medium at 28 °C with 5% CO_2_ for two hours. During DENV infection, cells were gently shaken at 15 min intervals. Afterwards, the infectious medium in C6/36 cells was replaced with a low serum RPMI 1640 medium containing 2% FBS, 1× NEAA, 1% P/S, and 1mM sodium pyruvate, and incubated at 28 °C with 5% CO_2_. The culture medium was collected at day 4, 7, 11, and 14, and cell debris were removed by centrifugation with 9,000× *g* for 30 min at 4 °C. The supernatant of culture medium was aliquoted and stored at −80 °C.

### 4.3. Plaque Assay

BHK-21 cells were seeded in six-well plates (4 × 10^5^ cells/well) at 37 °C with 5% CO_2_. Cells were inoculated with DENV suspensions in 10-fold serial dilution by serum-free DMEM (Gibco, 12100, Grand Island, NY, USA). The cells were incubated at 37 °C with 5% CO_2_ for two hours and were gently shaken every 15 min. After removal of virus suspension, the cells needed to be overlaid with 1:1 mixture of 2% low-melting agarose (Lonza, 50100, Rockland, ME, USA) with two-fold DMEM containing 4% FBS and 2% P/S. The infected BHK-21 cells were incubated at 37 °C with 5% CO_2_ for 7 days. Cells were fixed with 10% formaldehyde for 2 h post-infection. Next, the agarose was removed and the cells were stained with crystal violet for 4 h. Finally, the titer of the virus was determined by counting the numbers of the virus plaques.

### 4.4. Immunofluorescence Staining and Flow Cytometry Analysis

Sample cells were collected with the same density and washed with a staining buffer (PBS with 1% FBS). After centrifugation at 112× *g* for 5 min, the cells were re-suspended in a 100 μL staining buffer and stained with a 10 μL PE-conjugated anti-human TIM-1 antibody (R&D systems, clone#219211, Minneapolis, MN, USA) at 4 °C for 30 min. The negative controls were the unstained group (without antibody) and the isotype group, in which the cells were stained with the PE-conjugated mouse IgG2B (R&D systems, clone#133303, Minneapolis, MN, USA). Cells were washed with a 200 μL staining buffer twice, re-suspended in a 500 μL staining buffer, and temporarily stored at 4 °C. Cells were analyzed using a flow cytometer (Beckman coulter, CytoFLEX flow cytometer, Brea, CA, USA).

### 4.5. siRNA knockdown

Huh7.5 or GFP-LC3-Huh7.5 cells were seeded in a six-well plate with a density of 2 × 10^5^ at 37 °C, 5% CO_2_ overnight. The condition of siRNA transfection followed the protocol from the Lipofectamine RNAiMAX transfection procedure (Invitrogen, Lipofectamine RNAiMAX Reagent, Carlsbad, CA, USA). The amounts of siRNA in each well were 20 pmol. Human TIM-1 siRNA (ON-TARGET plus siRNA pool, Dharmacon, L-019856-00-0005, Lafayette, CO, USA) (si-TIM-1) and human PI3KR1 siRNA (ON-TARGET plus siRNA pool, Dharmacon, L-003020-00-0005, Lafayette, CO, USA) (si-p85) are designed to target TIM-1 and p85 mRNAs, respectively. Non-targeting siRNAs (si-control) are negative control pool of four siRNAs designed and microarray tested for minimal targeting of human, mouse, or rat genes (siGENOME Non-Targeting Control siRNAs, Dharmacon, D-001810-10-05, Lafayette, CO, USA). After knockdown 48 h, the knockdown cells were removed with trypsin and were seeded in a new six-well plate for autophagy activation or DENV production and infectivity experiments.

### 4.6. Preparation of Atto647N-Labelled DENV Particles

Virus-containing medium was centrifuged at 9,000× *g* for 30 min to remove cell debris and DENV particles were pelleted by ultra-centrifugation at 47,000 rpm in Beckman 50.2Ti rotor for 3.5 h. Virus pellets were resuspended in HNE buffer (5 mM HEPES, 150 nM NaCl, and 0.1 mM EDTA, pH 7.4) and further concentrated by ultrafiltration spin columns (GE healthcare, Cat. No. 28-9323-19, Little Chalfont, UK). Concentrated DENV particles were labeled with Atto647N-NHS ester (Sigma-Aldrich, Cat. No. 18373, St. Louis, MO, USA), with a maximum absorption at 646 nm and the maximum emission at 664 nm, to visualize individual DENV particles by a confocal microscope. Briefly, 2 × 10^7^ PFU of DENV were mixed with 4 nmol of Atto647N-NHS ester dissolved in dimethyl sulfoxide in an HNE buffer (5 mM HEPES, 150 nM NaCl, and 0.1 mM EDTA, pH 7.4) at room temperature for 60 min. The unincorporated dye was separated through a Sephadex G-25 column (GE healthcare, Cat. No. 17-0851-01, Little Chalfont, UK) and the eluted fractions were collected into different 1.5 mL tubes. The fractions containing Atto647N-labeling DENV were detected by a multimode microplate reader (TECAN 200/200Pro, Männedorf, Switzerland).

### 4.7. Confocal Microscopy Imaging

For immunofluorescent imaging, Huh7.5 or GFP-LC3-Huh7.5 cells were seeded in a 3.5 cm glass-bottom dish (Mettek) with a cell amount of 2 × 10^5^ and incubated at 37 °C with 5% CO_2_ overnight. Cells were washed with PBS, and then fixed with 4% paraformaldehyde at room temperature (RT) for 15 min. After fixation, cells were penetrated with 0.2% Triton X in PBS at RT for 10 min. The penetrated cells were soaked with a 1 mL blocking buffer (3% BSA in PBS, filtered with 0.45 mm filter) on a nutator at RT for 1 h. After washing with PBS, the cells were stained with a primary antibody on a nutator at RT for 4 h. After washing with PBS three times, the cells were stained with a secondary antibody on a nutator at RT for 2 h. The monoclonal Mouse anti-TIM-1 IgG (R&D systems, clone#219211, Minneapolis, MN, USA) was used to recognize TIM-1 and stained by Alexa Fluor532-conjugated anti-mouse IgG (Invitrogen, A-11002, Eugene, OR, USA). The monoclonal rabbit anti-p85 IgG (Abcam, ab191606, Cambridge, MA, USA) was used to recognize p85 and stained by Alexa Fluor647-conjugated anti-rabbit IgG (Invitrogen, A-27040, Eugene, OR, USA). Finally, the cells were stained with 4′,6-Diamidino-2-Phenylindole, Dihydrochloride (DAPI, Invitrogen, D1306, Eugene, OR, USA), washed with PBS three times, and kept in PBS for confocal imaging.

For DENV entry imaging, cells were seeded in a 3.5 cm glass-bottom dish and incubated overnight. Atto647N-DENV at MOI of 10 was resuspended in serum-free DMEM and inoculated in cells at 4 °C for 30 min. Next, cells were placed into a growth medium, and incubated at 37 °C, 5% CO_2_. At each time point, DENV-infected cells were washed with PBS and fixed with 4% paraformaldehyde for 15 min at RT. After washing with PBS, cells were kept in PBS for confocal imaging. Fluorescence images were acquired by confocal microscopes (Olympus, FluoView 1000 confocal microscope equipped with 100×, oil, NA1.4 objective lens, and Olympus, FluoView 10i confocal microscope equipped with 60×, oil, NA1.35 objective lens, Tokyo, Japan).

### 4.8. Quantification of DENV-Induced Autophagy by GFP-LC3 Puncta Numbers

GFP-LC3-Huh7.5 cells were seeded in a 6-well with a density of 2 × 10^5^ and were incubated at 37 °C with 5% CO_2_ overnight. The culture medium was removed and the cells were washed with PBS, followed by inoculation with DENV at MOI of 5 in serum-free DMEM (Gibco, 11995) at 4 °C for 30 min to allow DENV to binding on the cell surface rather than entering the cells. Cells were gently washed with PBS, and were shifted to 37 °C with 5% CO_2_ for DENV infection at different time points: 0, 15, 30, and 60 min. At each time point, the virus supernatant was removed and cells ware fixed with 4% paraformaldehyde at RT for 15 min. After being washed with PBS, the cells were stained with DAPI at RT for 10 min and the residual DAPI was cleaned with PBS twice. Cells were kept in PBS for fluorescence imaging. Fluorescence images were captured by Olympus IX70 equipped with 20× objective lens, and GFP-LC3 puncta numbers over 5 in one cell were regarded as a criterion for an autophagy positive cell. We determined the total cell numbers by recognizing the nucleus with DAPI staining and the numbers of autophagy positive cells. The percentage of autophagy positive cells was calculated by (autophagy positive cells/total cells) times 100%. The average percentages in autophagy positive cells were collected from 3 independent experiments and the fold change was determined by the average percentages of the DENV infection groups divided to that in mock group.

### 4.9. DENV Production Assay

GFP-LC3-Huh7.5 cells were seeded in a 6-well with a density of 2 × 10^5^ at 37 °C, 5% CO_2_ overnight. Pretreatment of siRNA in cells at 37 °C, 5% CO_2_ for 48 h. The cells were washed with PBS, inoculated with DENV at MOI of 1 at 4 °C for 30 min and shifted to 37 °C. At 24 or 48 h post-infection, the cell culture medium was collected and the extracellular virus titers were measured by a plaque assay.

## 5. Conclusions

In conclusion, we demonstrated that TIM-1 activates DENV-induced autophagy in the early phase of infection by recruiting p85 to virus-containing Rab5-positive endosomes. Our studies not only confirm TIM-1 as a DENV entry receptor, but also uncover a novel role of TIM-1 as a signaling receptor to trigger autophagy, resulting in facilitating the process of DENV life cycle.

## Abbreviations

AMPK	AMP-activated protein kinase
BHK-21	Baby hamster kidney-21
DENV	Dengue virus
DF	Dengue fever
DHF	Dengue hemorrhagic fever
DSS	Dengue shock syndrome
EBOV	Ebola virus
ER	Endoplasmic reticulum
HAV	Hepatitis A virus
HAVCR1	HAV cellular receptor 1
HCV	Hepatitis C virus
HSV-1	Herpes simplex virus-1
IKK	IκB kinase
JEV	Japanese encephalitis virus
LC3	Microtubule-associated protein light chain 3
MARV	Marburg virus
MFI	mean fluorescence intensity
MHV68	Murine gamma-herpesvirus 68
MILIBS	Metal ion-dependent ligand binding site
MOI	Multiplicity of infection
NS4A	Non-structure protein 4A
NS4B	Non-structure protein 4B
ZIKV	Zika virus
PI3K	Phosphoinositide 3-kinase
PtdSer	Phosphatidylserine
TIM-1	T-cell/transmembrane immunoglobulin and mucin domain protein-1
WNV	West Nile virus
PE	Phycoerythrin
